# Analysis of Cellulose and Lignocellulose Materials by Raman Spectroscopy: A Review of the Current Status

**DOI:** 10.3390/molecules24091659

**Published:** 2019-04-27

**Authors:** Umesh P. Agarwal

**Affiliations:** USDA, Forest Service, Forest Products Laboratory, Madison, WI 53726, USA; uagarwal@fs.fed.us

**Keywords:** polymorphy, crystallinity, supramolecular structure, nanocellulose, nanocomposite, lignin, syringyl, hemicellulose, non-linear, density functional theory

## Abstract

This review is a summary of the Raman spectroscopy applications made over the last 10 years in the field of cellulose and lignocellulose materials. This paper functions as a status report on the kinds of information that can be generated by applying Raman spectroscopy. The information in the review is taken from the published papers and author’s own research—most of which is in print. Although, at the molecular level, focus of the investigations has been on cellulose and lignin, hemicelluloses have also received some attention. The progress over the last decade in applying Raman spectroscopy is a direct consequence of the technical advances in the field of Raman spectroscopy, in particular, the application of new Raman techniques (e.g., Raman imaging and coherent anti-Stokes Raman or CARS), novel ways of spectral analysis, and quantum chemical calculations. On the basis of this analysis, it is clear that Raman spectroscopy continues to play an important role in the field of cellulose and lignocellulose research across a wide range of areas and applications, and thereby provides useful information at the molecular level.

## 1. Introduction

Raman spectroscopy, a label free spectroscopic method, was first applied to cellulose materials in the early 1970s [1,2,3,4] and subsequently, to lignin containing materials in mid 1980s [5,6,7]. Although at that time laser-excitation of the samples was limited to the visible region, fluorescence was a significant problem. Whereas for the latter, the presence of impurities in a cellulose sample was responsible, for lignin containing materials the sample itself was to be blamed. Additionally, due to single channel detection, the spectral acquisition period was too long. This situation improved gradually, over a span of 15 years, as improved instrumentation and longer wavelength lasers for sample excitation became available. For instance, in the 1990s new technologies like the holographic notch filter and the availability of charge-coupled devices (CCD) that acted as multichannel detectors decreased acquisition time by more than an order of magnitude. Rugged, air-cooled lasers (e.g., He–Ne 633 nm) simplified utility requirements and provided more beam-pointing stability compared with that of water-cooled lasers. Furthermore, sampling in confocal mode reduced sample fluorescence by physically blocking the signal originating from the volume of the sample not in focus. The detected Raman signal came from the illuminated spot. These capabilities permitted compositional mapping of the woody tissue (a lignocellulose material) with chosen lateral spatial resolutions. Thousands of spectra could now be obtained in a practical way.

Similarly, especially for milled-wood lignin and lignocelluloses, the problem of sample fluorescence could be largely avoided with the availability of the 785 and 1064 nm lasers as excitation sources. Although, at such excitation wavelengths, native lignin generally does not fluoresce, industrial lignins still do. Consequently, investigations of certain lignins remain limited in linear/traditional Raman spectroscopy.

There are still other factors that continue to play a role in the growth of Raman spectroscopy applications. In the present context, these have to do with (1) continued investigations of plant tissues, (2) applications to materials based on nanocelluloses, (3) development of superior high-resolution techniques for Raman imaging including linear and nonlinear Raman microscopy, (4) advances in the design and control of ultrafast lasers for applications exploiting nonlinear Raman processes, and (5) developments in chemometric analysis of Raman data. All these advances have made Raman spectroscopy an essential analytical technique that permits probing of cellulose and lignocellulose materials at the molecular level in a variety of matrices and sampling environments.

Because this review is limited to last 10 years or so, for those interested in the earlier work, there are a number of reviews available [8,9,10,11,12,13,14,15]. It is hoped that this review focused on the field of celluloses and lignocelluloses will serve as a status report on the use of Raman spectroscopy and simultaneously, highlight of some of the many applications in the field.

## 2. Cellulose Materials

Cellulose, known as nature’s polymer, is the most abundant biomaterial on earth. Cellulose materials are largely used in food, materials, medical, and pharmaceutical industries. In the field of cellulose and lignocelluloses, cellulose and cellulose products (e.g., cotton, pulp, paper, and cellulose nanomaterials) along with cellulose derivatives are the materials most often investigated using Raman spectroscopy due to the fact that generally speaking these materials are not inherently fluorescent and therefore, spectra with good signal-to-noise ratio can be obtained. In the spectra of cellulose materials, most of the Raman features of cellulose have been identified and assigned [16]. However, although most of the vibrational modes are highly coupled due to cellulose chain consisting of C–C and C–O bonds, better band assignment still needs to take place for some of the bands [17].

### 2.1. Characteristic Bands

Characteristic Raman bands of cellulose in various materials have been reported. A few of these studies consisted of investigations of Valonia ventricosa [1], Valonia macrophysa and Ramie [16], cotton [18], and plant cell wall cellulose [19]. While for a large number of bands, the band positions between such substrates were similar, band intensities and profiles (e.g., band widths) differed significantly. Such differences are largely due to variances in the supramolecular structures (mostly, crystallinity) of aggregated states of cellulose (more later). Such basic information is essential in the interpretation of spectral data from cellulose materials.

### 2.2. Allomorphs of Cellulose

**Figure 1:**
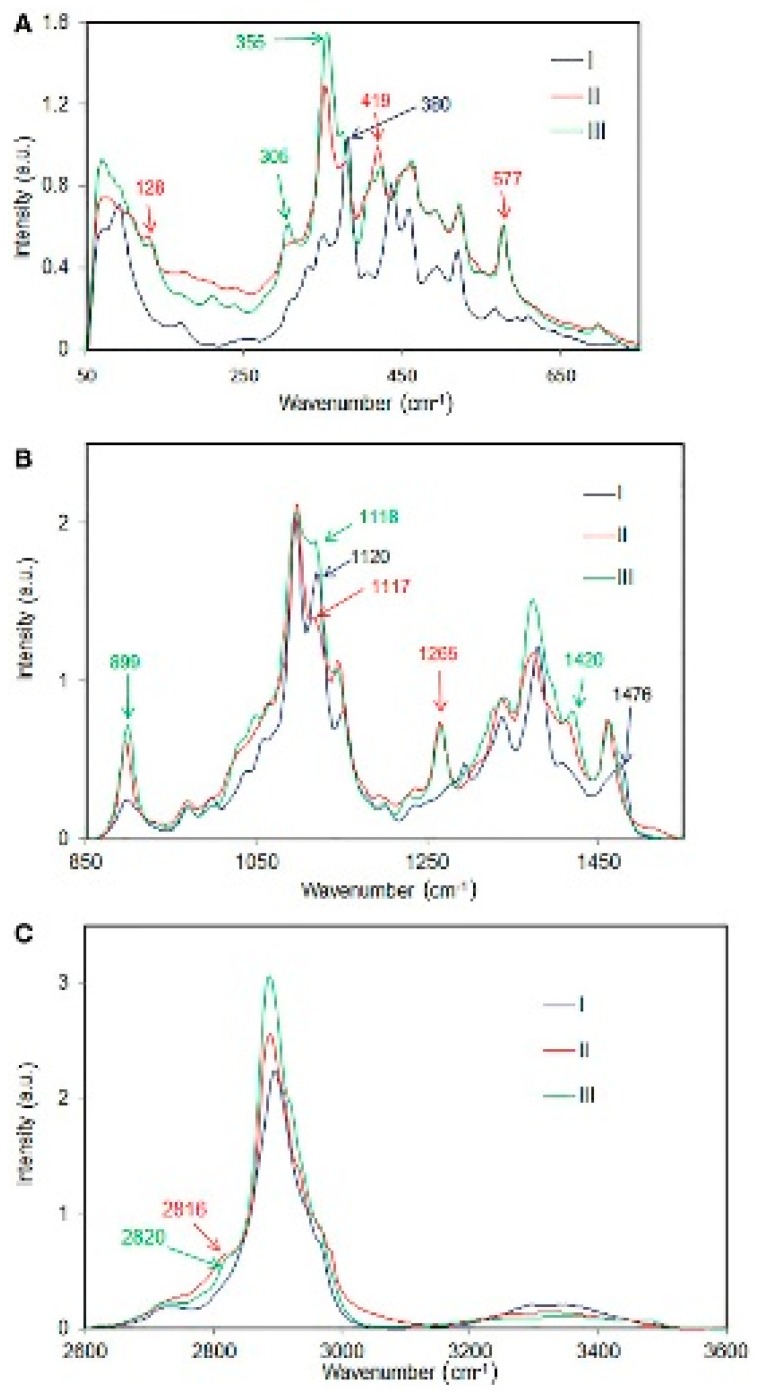
Comparison of Raman spectra of cellulose I, cellulose II, and cellulose III in various spectral regions; (**A**) 50–750 cm^−1^, (**B**) 850–1550 cm^−1^, (**C**) 2600–3600 cm^−1^. Reproduced from Ref. [13].

Cellulose molecules can aggregate in a wide variety of secondary and tertiary structures. This is brought about by variations in the intra- and inter-molecular hydrogen bonding and the organization of the cellulose chains (e.g., parallel vs. antiparallel or chain polarity). These aspects influence the crystal parameters of the various allomorphs (crystalline phases) which in turn dictate the overall structural accessibility (e.g., during mechanical, chemical and enzymatic treatments), and the reactivity of cellulose. There are at least six cellulose polymorphs reported in the literature—cellulose I, cellulose II, cellulose III_I_, cellulose III_II_ cellulose IV_I_ and cellulose IV_II_. However, some researchers think that cellulose IV is not a pure allomorph but rather a mixture of cellulose I and cellulose II. Moreover, it was reported that cellulose I is a composite of cellulose Iα and Iβ crystalline forms. Raman spectroscopy has been used to study these allomorphs and also to differentiate between them when present in a material together. Specific Raman bands associated with allomorphs cellulose I, cellulose II, and cellulose III have been reported [4,13] and in the cases of a mixture of cellulose I and cellulose II and cellulose I and cellulose III it was reported that amounts of the allomorphs can be quantified by Raman spectroscopy [20,21]. In Figure 1 below, Raman spectra of cellulose I, cellulose II, and cellulose III are compared [13].

From the polymorphic sensitive information in Raman, materials that contain more than one type of crystal/aggregated state can be characterized and the various forms present quantified. Therefore, physicochemical properties of cellulose materials can be understood and improved upon for applications.

### 2.3. Cellulose Crystallinity Measurements

**Figure 2:**
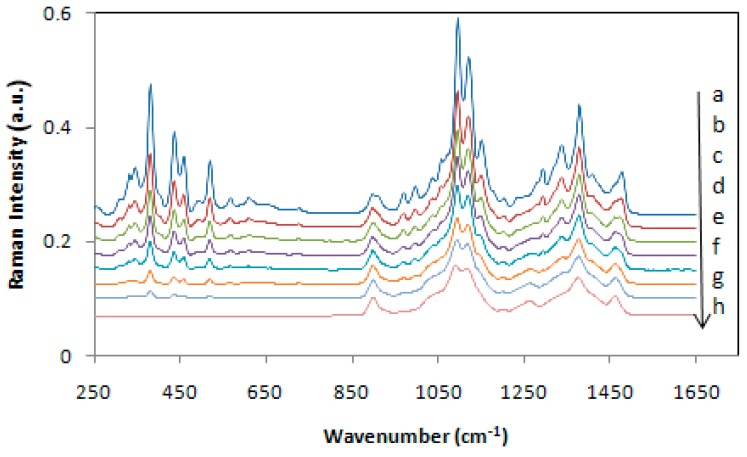
Calibration set Raman spectra after subtracting amorphous spectrum in the region 250–700 cm^−1^; (a) control, cotton microcrystalline cellulose, and plots (b) to (h) are spectra of mixture 1, mixture 2, mixture 3, mixture 4, mixture 5, mixture 6, and 120-min ball milled cellulose, respectively. Note that in the case of 120-min spectrum the intensities below 700 cm^−1^ are all zero because of the subtraction. Spectra were offset on the intensity scale for display purposes. Reproduced with permission from Ref. [23]. Copyright Springer Nature 2010.

**Figure 3:**
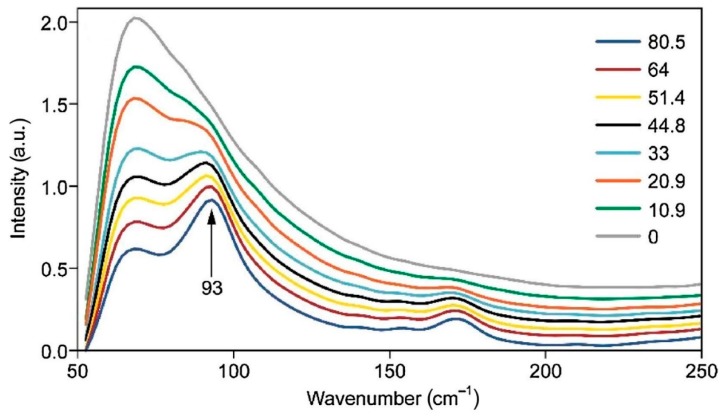
Low frequency Raman spectra of calibration set samples, calculated crystallinities of the samples are listed on the left hand side in the Figure. Reproduced with permission from Ref. [24]. Copyright Springer Nature 2010.

Although Raman spectroscopy has been used for a long time for qualitative analysis of celluloses and cellulose materials, it was not until 2005 that a method was proposed to measure cellulose crystallinity [22]. The method was based on the CH2 deformation modes (1450–1480 cm^−1^). However, the method requires separation of the contributions of amorphous and crystalline forms of cellulose, and for that, a deconvolution process is used. However, the deconvolution methodology has its own limitations. Subsequently, two more Raman methods, one in 2010 [23] and the other in 2018 [24] were published. Both the methods are free of the deconvolution requirement and use low wavenumber Raman bands (380 and 93 cm^−1^) to estimate cellulose crystallinity. Compared to the 380 cm^−1^ based method (380-Raman), the advantage of the 93 cm^−1^ based method (93-Raman) is that it differentiates between the crystalline and organized cellulose, the latter being an aggregated form of cellulose that is oriented and aligned but at the same time, not-crystalline. Nevertheless, for the 93 cm^−1^ peak based method, considering the difficulty of performing low frequency Raman due to both scattering and sample fluorescence issues, an FT-Raman instrument with 1064 nm excitation is needed. In non-FT spectrometers, a strong contribution from Rayleigh scattering masks the low frequency (LF) Raman scattering from the samples. In Figure 2 and Figure 3 the Raman spectra of the calibration set samples are shown, respectively for 380-Raman (23) and 93-Raman (24) methods. The samples, mixtures of the cotton microcrystalline cellulose and completely amorphous cellulose, were used to develop the crystallinity methods. Figure 2 below shows Raman spectra of a set of cellulose samples that were used to develop the calibration for the two Raman methods in our laboratory, namely, 380-Raman (Figure 2) [23] and 93-Raman (Figure 3) [24].

Because crystallinity has an important effect on the physical, mechanical, and chemical properties of cellulose (e.g., with increasing crystallinity, tensile strength, dimensional stability, and density increase, while properties such as chemical reactivity and swelling decrease), its accurate determination is very important. Compared to some cellulose crystallinity methods, the 380-Raman [23] and 93-Raman [24] methods have unique advantages and produce reliable data. Availability of such reliable information is useful in predicting the applicability of the cellulose materials.

### 2.4. Characteristics of Supramolecular Structures

Crystalline cellulose is one manifestation of how cellulose molecules are organized at the tertiary or 3-dimensional level and is measured by estimating cellulose crystallinity. Yet other measures, some of them based on Raman spectroscopy, exist to characterize the other aggregated states of cellulose. For instance, supramolecular structure of cellulose in native state in woods, which was found to be non-crystalline, has been defined by its accessibility to water (based on Raman intensity increase at 1380 cm^−1^ upon sampling in D_2_O vs. H_2_O) [25]. This Raman spectroscopy determined parameter, was found to be related to be correlated with degree of lateral order (DOLO) which is based on FWHM (full-width at half maximum) of [200] peak in X-ray diffraction [25]. A second Raman spectroscopic measure, is the ratio of the peak heights of bands at 1460 and 1480 cm^−1^ [25]. In the aggregated state, this indicates the extent of disorder in cellulose that exists at C6 at the intra-molecular level. The two Raman bands at 1460 and 1480 cm^−1^ represent the deformation modes of the methylene groups of the exocyclic CH_2_OH group. In light of these two Raman parameters, non-crystalline aggregated states of cellulose that are not easily described can now be characterized. This is an unparalleled capability of Raman spectroscopy and is expected to play an important role in characterizing the supramolecular structures of cellulose in various materials.

### 2.5. Nanocelluloses

**Figure 4:**
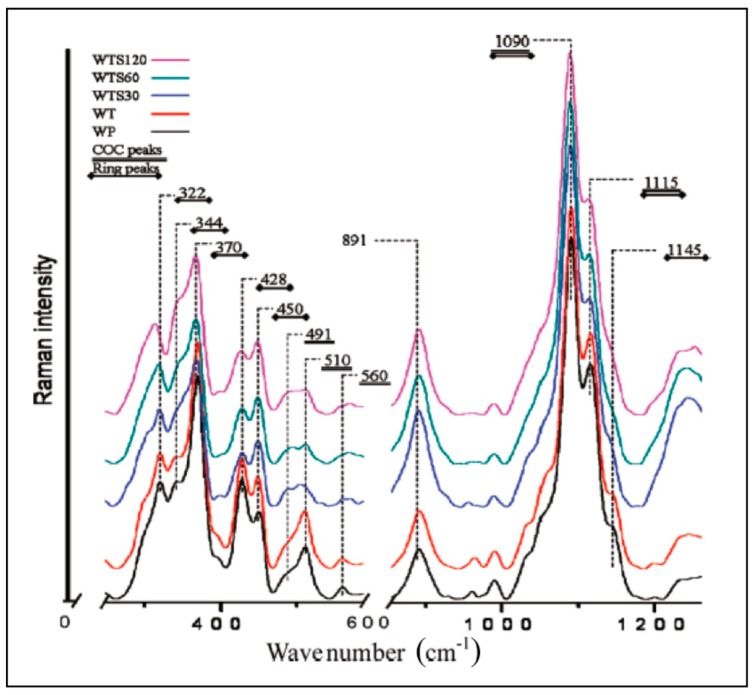
Raman spectra of molecularly thin (single digit angstrom thickness) cellulose nanoparticles that were obtained by intensive sonication of TEMPO-oxidized cellulose fibers. Spectra of TEMPO treated (WT) and control wood pulp (WP) are also shown. Reproduced with permission from Ref. [30]. Copyright American Chemical Society 2011.

When it comes to analyzing cellulose nanomaterials (CNs—cellulose nanofibrils and cellulose nanocrystals), Raman spectroscopy is uniquely suited because, these materials can be analyzed in their native hydrated states without any special considerations. In our work on CNs, we have used Raman spectroscopy for estimation of crystallinity (in suspensions and freeze-dried states) [20,26,27,28], measurement of accessibility of the nanomaterials by water [20,26], detection and quantitation of cellulose II polymorph in the nanomaterials [20], and effect of drying on the structure of CNs [20]. Moreover, because Raman spectra of the nanomaterials contain bands that are associated with chemical functionalities usually present on the surfaces of prepared/modified materials (for example, sulfate esters present on the surfaces of the sulfuric acid produced CNCs) they can be detected and quantified. For instance, trans esterified CN, where surface hydroxyl groups were esterified, was characterized by Raman spectroscopy [29]. Yet another application was in the area of crystallinity determination of the cellulose nanofibrils that were disintegrated by various processing approaches (refining and microfluidization) [28]. In another application, Raman spectroscopy was used to characterize supramolecular structure of molecularly thin cellulose I nanoparticles [30]. Raman spectra from molecularly thin cellulose nanoparticles (WTS30, WTS60, WTS120), control wood-pulp (WP), and TEMPO ((2,2,6,6-Tetramethylpiperidin-1-yl)oxyl) treated wood pulp (WT) are shown in Figure 4. In the nanofibrils, clear changes in the cellulose’s supramolecular structures were noted based on the spectral analysis.

CNs are derived from natural resources and Raman spectroscopy plays a significant role in various aspects of their R & D (production, properties and applications), so that novel and advanced materials from the CNs can be developed.

### 2.6. Cellulose Nanocomposites

Confocal Raman microscopy is being increasing applied to study composites of CNs [31,32,33,34,35,36]. Typically, the nanocomposites consist of thermoplastics and CNs. Additionally, composites of cellulose nanofibrils and cellulose nanocrystals have also been investigated (author’s unpublished work). In most instances, confocal Raman microscopy is used to investigate how well CNs are distributed in a composite because it has been reported that aggregation of the CNs, which are the load bearing component in a thermoplastic composite, inhibits the stress-transfer process. Because CNs are hydrophilic, they tend to aggregate and are not evenly distributed. One of the earlier investigations on this topic focused on composites cellulose nanocrystals (CNC)-polypropylene [31]. Such analysis showed that CNCs were aggregated to varying degrees in the composites and remained poorly dispersed in the polypropylene matrix. A recent study was performed to evaluate quantitatively distribution of CNCs in high-density polyethylene (HDPE) composites [33]. The Composites were prepared using maleic anhydride modified polyethylene (MAPE) and poly(ethylene oxide) (PEO) as a compatibilizer. The researchers reported that it was possible to quantify the distribution and mixing of cellulose nanocrystals (CNCs) in a polyethylene-matrix composite.

To understand how stress is transferred from matrix to CNs in a composite, Raman spectroscopy has been used. It has been reported that under mechanical tension the cellulose band at 1095 cm^−1^ shifts to lower frequencies [34]. This characteristic of the Raman band has been used by researchers to study what influences the stress transfer in various composites. For example, Rusli et al. [35] investigated tunicate CNC and poly(vinyl acetate) nanocomposites by polarized Raman spectroscopy and showed that the stress transfer was influenced by local orientation of the nanocrystals. Shifts of the 1095 cm^−1^ band as a result of uniaxial deformation of nanocomposite films were used to determine the degrees of stress experienced by the CNCs, not only due to stress transfer from the matrix to the tunicate CNCs but also between the CNCs within the composite. In another case, short cellulose nanofibrils (SCNF) were used as reinforcement in polyvinyl alcohol (PVA) fiber and the system was analyzed by Raman spectroscopy [36]. The researchers reported that the strength and modulus of PVA/SCNF composite fiber with a SCNF weight ratio of 6 were nearly 60 and 220% higher, respectively than that of PVA by itself. Shifts in the Raman peaks at 1095 cm^−1^ indicated good stress transfer between the SCNF and the PVA matrix.

Therefore, both at the macro and sub-micron levels, Raman analysis gives beneficial information, on the topics in nanocomposites research where further development needs to take place to improve the compatibility between CNs and the matrix. Once such problems are successfully addressed, practical applications of the nanocomposites will ensue.

## 3. Lignocellulose Materials

Cellulose materials that also contain hemicellulose and lignin are called lignocelluloses. Such materials are abundantly available for multiple uses including biofuels production. Lignin is an aromatic polymer whereas both cellulose and hemicellulose are carbohydrate polymers. Lignin is found in the cell walls of vascular plants. Raman spectroscopic applications to lignin containing materials had a slow start largely due to the fact that significant fluorescence from lignin was generated upon excitation by visible lasers. Quenching of this fluorescence was difficult because it was caused by one of the components of the material and not by an external impurity, as is the case with cellulose. Although with the advent of confocal Raman microscopy this situation improved somewhat because the technique limited fluorescence by physically blocking the signal originating from the locations other than the sample in focus. Moreover, sampling in water and in an environment of molecular oxygen helped—most likely, due to degradation/quenching of the fluorescence caused by chromophores in lignin. Later, in mid 1990s, with the availability of the 1064 nm near-IR laser for sample excitation, the fluorescence problem was mostly eliminated since not many lignocellulose materials fluoresced at such a long wavelength of excitation [8,9,19]. Nevertheless, analysis of industrial lignins continues to be a challenge in linear/ordinary Raman spectroscopy due to the fact that such dark colored lignins generate extensive amount of fluorescence even at 1064 nm excitation.

Early work on lignocelluloses started with studying the ultrastructure of wood, especially orientation of lignin in wood cell walls. Subsequently, role of lignin in high yield pulp yellowing (thermal and photo) was investigated. Later on, efforts to quantitate lignin in woods were made but failed due to the fact that aromatic ring conjugated substructures in lignin disproportionately contributed to the intensity of 1600 cm^−1^ band (pre-resonance Raman and conjugation effects). However, once such structures were removed, for instance in unbleached kraft pulps, residual lignin could be quantified [37]. Other wood-pulp studies made use of resonance Raman spectroscopy to avoid the problematic fluorescence. FT-Raman spectroscopy was applied for classifying woods into hardwoods and softwoods. For more information on past work related to lignin, a recent review nicely covered the applications of Raman spectroscopy to understand the lignin containing biomass [14] and its processing. Similarly, a review of Raman microscopy applications to understand plant cell walls and their structure-function relationships has been provided [15]. Some other confocal Raman microscopy work focused on the altered lignin structure of CAD (cinnamyl alcohol dehydrogenase) deficient transgenic poplar [38], study of lignin distribution in Eucalyptus cell walls during GVL/water/acid treatment [39], identification of hemicellulose features in poplar cell wall (in conjunction with multivariate analysis) [40], effects of weathering on wood surfaces [41], and characterization of ionic liquid swelled cell walls [42,43,44].

### 3.1. Lignin Bands

All plant lignins are composed of three types of aromatic nuclei—guaiacyl (G), syringyl (S) and p-hydroxyphenyl (H). In Raman spectra of lignocellulose materials, lignin bands have been identified in woods (both softwoods and hardwoods) and sugarcane bagasse (pith)—a grass. Because there are many structural differences not only between lignins of the plants but also between lignins’ compositions, lignin Raman spectra vary between lignocellulosics and also within a particular type of lignocellulose (e.g., woods; angiosperms vs. gymnosperms). Lignins can be classified as G-lignins (from softwoods), GS-lignins (from hardwoods), GSH-lignins (from grasses) or GH-lignins (from compression wood). Wood lignins have been studied the most either in the native state [15,19] or upon isolation as milled-wood lignins [19,45]. The Raman bands of softwood and hardwood lignins have been assigned in literature although some of the assignments continue to be a topic of ongoing research. The assignment work is largely based on the studies of lignin models [46,47] and the refinements that have been made using DFT (density functional theory) calculations [48]. From the models used in the work (4-methylphenol, 2-methoxy-4-methylphenol, and 2,6-dimethoxy-4-methylphenol), the DFT refinements were made on characteristic H, G, and S bands and related to specific vibrations based on the DFT calculations.

### 3.2. Spectral Domination by the Conjugated Structures in Lignins

Early on, when the lignin quantitation work based on Raman spectroscopy did not succeed, it was recognized that some of the subunits in its structure contributed disproportionately to the observed scattering at 1600 cm^−1^. Subsequent work involving 19 lignin model compounds established that in conventional Raman spectroscopy the intensity enhancement in the spectrum was due to both pre-resonance Raman and conjugation effects [8,9]. Comparatively, in near-IR Raman using forty models, it was established that only conjugation effect contributed to the increased intensity [46]. Therefore, quantitation is not straightforward and only possible if same lignin structure is assumed in all samples [13]. This is exemplified by the Raman study of a number of lignocellulose materials where the contributions to 1600 cm^−1^ band by the lignin conjugated structures were removed/reduced by treatment with alkaline hydrogen peroxide (H_2_O_2_), diimide (N_2_H_2_), and sodium borohydride (NaBH_4_) treatments [13,45]. This meant that in cases where lignin structure is non-homogeneous, lignin quantitation is achievable using Raman spectroscopy. The advantage of Raman spectroscopy in being sensitive to the conjugated lignin substructures is that such structures can be easily detected when present in small quantities. An example is the detection of pinosylvins (a kind of wood extractive) in Scots pine wood by Raman spectroscopy [49]. In Table 1 below, for black spruce milled-wood lignin (MWL), data is shown that shows the manner in which Raman band positions/intensities change upon various chemical treatments [45].

### 3.3. Differentiation between G-, S-, and H-Lignins

When one considers the Raman spectra of the G, S, and H lignins, certain Raman spectral differences stand out. These have been described in various publications. In the case of S-lignin spectrum, bands at 2940, 1455, 1331, 1156, 1037, 597, 531, 522, 503, 472, 447, 431, 417, and 369 cm^−1^ either are more intense compared to G-lignin bands or are only present in its spectrum [45]. Similarly, in G-lignin’s case, such bands are present at 1298, 557, and 384 cm^−1^ [45]. Such differences are important in further developing Raman spectroscopy for applications to investigate lignin and lignocellulosics. For instance, % syringyl groups in lignin (%S) have been quantified using Raman spectroscopy (see below). Although lignins dominant in H units seem to have not been investigated by Raman spectroscopy, H Dehydrogenation Polymer (DHP) lignin has been investigated [50]. Based on this work, compared to the syringyl and guaiacyl DHPs, the H-DHP showed weak bands in the aliphatic C-H stretch region. This is likely to be due to the fact that in these DHPs there were not many lignin units with -OCH_3_ groups. Additionally, compared to the spectra of S and G DHPs, the bands at 370, 600, 730, 820, 904, 1035, 1100, 1150, 1366, 1456, 1508 cm^−1^ were missing. However, new bands were detected at 831, 1173, 1257, 1393 cm^−1^.

### 3.4. %S Content and S/G Ratio

Lignin plays a significant role in the growth and development of plants and at the same time, has to be dealt with in the industrial utilization of lignocellulose biomass. The lignin monomer composition and, therefore, its %S content is an important parameter for lignocellulose biomass characterization and utilization. Various techniques, both wet-chemical and spectroscopic methods have been used to estimate the H, G, and S monomer composition of lignins. Previously, Raman spectroscopy in conjunction with thioacidolysis has been used to develop PLS (partial least squares) models for predicting lignin S/G ratios [51]. However, this multivariate approach lacks simplicity and specificity and involves first developing a predictive model based on a large number of samples. In another FT-Raman approach [52] to determine lignin S/G, first, S and G bands in the spectra of lignin models were identified. To separate S and G contributions in the spectra of lignocellulosic substrates, spectral deconvolution between 1220 and 1530 cm^−1^ was carried out. By integrating the intensities of the deconvoluted Raman bands that represented the S and G units the Raman S/G ratio was calculated. Next, a regression model was constructed between the FT-Raman and pyrolysis-GC/MS S/G ratio results and was used to estimate final S/G ratios [52]. However, sample spectral regions assigned to S, G, and H lignin units had significant interference not only due to sample fluorescence, but also from overlap with cellulose and hemicellulose contributions. Moreover, it is well known that the deconvolution approach used is a subjective method, and the obtained values may not be reliable. More recently, the author’s laboratory has developed a technique that uses no such method [53]. This Raman method is based on quantifying the intensity of the 370 cm^−1^ peak in wood lignins. This band in the Raman spectra of hardwoods is known to be due to syringyl units in lignin [45].

## 4. Hemicellulose Contributions

The molecular structures of hemicelluloses (branched amorphous polysaccharides composed of different carbohydrate monomers, a mixed polymer) are similar to that of cellulose and therefore, in the Raman spectra, the two types of contributions overlap [19]. This was borne out by the earlier investigations where the Raman spectra of cellulose, glucomannan, and xylan were compared [19]. Nevertheless, because usually cellulose exists in an organized/crystalline state, its Raman features are much sharper and stronger compared to that of the hemicelluloses. More recently, Zhang et al. [40] reported Raman spectrum of hemicellulose in poplar cell wall that was based on multivariate analysis. As expected, in the simulated components spectra of hemicellulose and cellulose, there was significant overlap of Raman bands [40].

## 5. New Methods of Spectral (Data) Analysis

Most methods of analyzing complex set of Raman spectra fall in the category of Chemometrics. Chemometrics can be described as the application of mathematical and statistical methods to extract more useful information from chemical and physical measurement data. Within this field, subtopics of data preprocessing, genetic algorithms, spectral image processing, data compression, and calibration transfer are generally included. Using this approach, the spectral changes or differences are visualized with the help of multivariate statistical analysis (linear discriminant analysis, partial least squares regressions etc.). In such approaches, using baseline corrected spectra gives superior results. Moreover, in the case of irregularly shaped fluorescence background, truncating spectra first then BG correction gives better results compared to correction the whole spectrum at once. The multivariate methods are used in quantitative measurements because they can detect unanticipated sources of variance in the data. In the earlier days, Raman analysis was carried out in combination with the chemometric methods partial least squares (PLS) and principal component analysis (PCA). For example, PLS was used to build a predictive model for estimating cellulose crystallinity for cellulose materials [23]. Other methods were subsequently applied to plant cell walls [15,54,55,56]; complex multicomponent lignocellulose samples, that produced complex Raman spectra so that the identification and quantitation of individual constituents can be carried out. To automatically identify Raman spectra of different cell wall layers (cell corner—CC, compound middle lamella—CML, secondary wall—SW, gelatinous layer—G-layer, and cell lumen), Zhang et al. [54] proposed a new chemometric method on the basis of PCA and cluster analysis. Later on, Zhang et al. [40] applied a chemometrics technique called self-modeling curve resolution (SMCR) to poplar cell wall Raman imaging data to discriminate the spectral contributions of cellulose and hemicellulose to demonstrate how the two components were distributed in various cell types of the woody plant. Similarly, Prats-Mateu et al. [56] applied vertex component analysis (VCA), non-negative matrix factorization (NNF) and multivariate curve resolution–alternating least squares (MCR-ALS) to gain insights into the spectra obtained as a result of Raman imaging of the plant cell walls. Vertex component analysis was recommended as a good preliminary approach whereas the other two tools had some negative aspects to them. It was reported [56] that while investigating spruce wood and Arabidopsis, although NMF, MCR-ALS, and VCA were used to find the purest components within the confocal Raman microscopy datasets, the endmember spectra obtained using VCA were more correlated and mixed than those retrieved by NMF and MCR-ALS methods. Moreover, it was stated that the former two methods can also lead to artificial band shapes due to peak splitting or inverted bands.

## 6. New Raman Techniques (Instrumentation)

As the instrumentation has evolved in the field of Raman spectroscopy, so has the ability of Raman spectroscopy to deliver useful information in the field of cellulose and lignocelluloses. Early on, the problem of fluorescence swamping the weaker Raman signal in the study of lignin containing materials was overcome by using a Kerr gated Raman collection system [57]. In this study, chromophore lignin structures in wood-pulp samples were analyzed. In the Kerr system, fluorescence is rejected in the time domain with the Kerr gate acting as an optical shutter. In one investigation, lignin radicals in the plant cell wall were probed by this technique [58].

**Figure 5:**
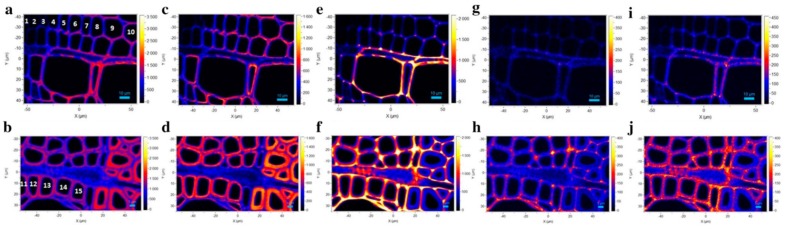
Raman images of xylem for poplar wood calculated by integrating from 2800 to 3030 cm^−1^ (**a**, **b** overall morphology), from 2800 to 2918 cm^−1^ (**c**, **d** carbohydrates), from 1540 to 1700 cm^−1^ (**e**, **f**, lignin), from 1255 to 1290 cm^−1^ (**g**, **h** G units), and from 1320 to 1338 cm^−1^ (**i**, **j**, S units). Images **a**, **c**, **e**, **g**, **i**: xylem near the cambium and images **b**, **d**, **f**, **h**, **j** xylem near the annual ring (AR). Reproduced with permission from Ref. [59]. Copyright SPRINGER NATURE 2018.

Confocal Raman imaging [12] where analysis is carried out at diffraction-limited spatial resolution level to obtain information on the chemical constituents and their distributions within the sample has been used quite widely to investigate tissues of various plants (e.g., stem, leaves, and roots). Additionally, different vascular cell types (e.g., xylem and phloem) in woody and herbaceous plants have been studied by this spectroscopy technique [59]. Plant cell walls are highly complex structures and during growth and development phase, undergo changes. For poplar wood xylem and phloem, the kind of information that can be generated is shown in Figure 5 [59]. Another area where Raman imaging has played a role is in the area of nanocellulose composites [31] where the technique has allowed a better understanding of the cellulose/polymer interface.

To deal with the sample fluorescence and small amounts of lignin substructures (e.g., in commercial and milled-wood lignins), surface-enhanced Raman spectroscopy (SERS) was used by Agarwal et al. [60]. In SERS, Raman scattering is enhanced of molecules adsorbed on rough metal surfaces (e.g., silver or gold) or by nanostructures such as plasmonic-magnetic silica nanotubes. The enhancement factor can be as much as 10^10^ to 10^16^, which means the technique may detect single molecules.

**Figure 6:**
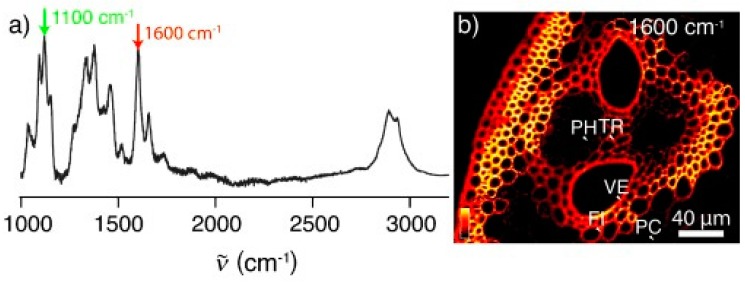
Raman spectrum and SRS imaging of corn stover. (**a**) Raman spectrum of raw corn stover. The peak at 1600 cm^−1^ (red arrow) corresponds to the lignin distribution, and the peak at 1100 cm^−1^ (green arrow) corresponds to cellulose. (**b**) SRS image of the vascular bundle including the edge of the stem in raw corn stover at 1600 cm^−1^, showing the lignin distribution. Labeled structures are discussed in the text: parenchyma (PC), phloem (PH), vessel (VE), tracheid (TR), fiber (FI). Reproduced with permission from Ref. [61]. Copyright JOHN WILEY AND SONS 2010.

In the context of producing biofuels (e.g., cellulosic ethanol), biomass processing has been monitored by stimulated Raman scattering (SRS) microscopy (Figure 6 below) [61,62]. In SRS, two laser beams at ωp and ωS coincide on the sample. When the difference frequency Δω = ωp − ωS (also called the Raman shift), matches a specific vibrational frequency, amplification of the Raman signal is achieved by virtue of stimulated excitation of molecular transition rate. Zeng et al. [62] reported that this technique can be readily applied to understand the pretreatment reaction, and can also be useful for studying the enzymatic breakdown of cellulose or for in vivo imaging of lignification and cellulose biosynthesis in plant cell-wall development. In another application of SRS, maleic acid pretreatment resulted in effectively removing of the loosely packed lignin in secondary walls which in turn led to enzyme accessibility for digestion of the biomass [62]. In yet another application, in conjunction with of enzymatic digestion, SRS technique was used by the researchers to establish that xylan can be resolved from cellulose and lignin [63].

In coherent anti-Stokes Raman spectroscopy (CARS) two beams of frequency ω_1_ and ω_2_ are mixed in the sample to generate a new frequency ω_s_ = 2ω _1_ − ω_2_. If there is a Raman resonance at ω_1_ − ω_2_ = Ω, an amplified signal is detected at the anti-Stokes frequency ω_1_ + Ω. Compared to conventional Raman, CARS offers greater sensitivity and rapid analytical capability. CARS microscopy, a multi-photon technique, has been applied to wild-type and lignin-downregulated alfalfa lines to assess the impact of lignin modification on overall cell wall structure [64]. In another work reported on cellulose fibers, CARS along with second harmonic generation (SHG) microscopy was used to investigate molecular alignment in dry and hydrated cellulose [65].

In the biofuels area, clearly from the examples cited above, Raman applications/analyses provided information that when factored in and successfully worked on the remaining problems will advance the field. Therefore, it contributes to generating solutions to the problems at hand.

## 7. Quantum Chemical Calculations

Although, to interpret experimental results, the use of quantum computational methods (typically using density functional theory, DFT) is standard practice in Raman spectroscopy, the methods have been only occasionally used in the cellulose and lignocellulose field. This may be because all the component biopolymers of plants (cellulose, hemicellulose, and lignin) give rise to many vibrational bands that are combinations of several vibrational modes which involve various atoms in the molecules. In addition to correlating the band positions with the molecular motions (normal modes), the DFT methods are capable of predicting intensities and bandwidths of the spectral features. This capability is therefore, adding to our ability to calculate and interpret Raman spectroscopic measurements. In the following a few example s are described where the quantum chemical methods have aided the spectral interpretation.

In 2006, Barsberg et al. [57] used DFT to correlate the resonance Raman spectroscopy results with the predicted vibrational modes of lignin radicals, and indicated that the radicals were formed from syringyl and guaiacyl moieties in beech and spruce, respectively. Similarly, in 2010, Raman bands of lignin model monomeric structures were compared with density functional theory predictions [47]. For analyzing vibrational modes of cellulose, DFT calculations with the cellulose Iα and Iβ structures were carried out [66,67]. Such calculations have resulted in not only the assignment of experimentally detected bands (e.g., assignments of the 380 and 93 cm^−1^ bands in cellulose) but also questioning of some of the earlier assignments (H-bonded OH modes) in cellulose. The assignments of the 380 and 93 cm^−1^ bands are particularly important because they have been identified as measures of cellulose crystallinity.

## 8. Conclusions and Outlook

In conclusion, the future of applying Raman spectroscopy to investigate cellulose and lignocellulosic materials is bright. The information provided by Raman spectroscopy is unique and obtained in situ. Past applications have ranged from understanding cellulose crystallinity and biomass recalcitrance to characterizing lignin and cellulose nanomaterials. Although it took quite a while for the Raman applications to arrive at this juncture and even now, in most laboratories, the technique has not become a regularly used analytical tool, going forward, it is likely that this powerful spectroscopy with its multitude of sub-techniques will be implemented by many more researchers. In the context of plant deconstruction and biofuels, Raman spectroscopy methods meet the need to screen rapidly and accurately large collections of different plant materials. Various Raman imaging techniques have allowed researchers to assess the biomass pretreatment processes in real-time, providing insights into how specific treatments affect the constituents of the biomass. Finally, future advancements in the field of Raman spectroscopy will continue to offer researchers an analytical capability that is a versatile, non-destructive, and user-friendly. Nevertheless, challenges remain in applying linear Raman spectroscopy to fluorescent materials (e.g., commercial lignins). However, non-linear techniques (e.g., SRS and CARS) are free of this problem although spectra of commercial lignins do not seem to have been reported in the literature.

## Figures and Tables

**Table 1:** Treated black spruce milled-wood lignins (MWLs)—Raman frequencies (cm^–1^) and change in peak-intensity of most intense untreated MWL bands. Reproduced with permission from Ref. [45]. Copyright TAYLOR & FRANCIS 2011.

Untreated	H_2_O_2_ bl. (% Δ Int.)	Hydro. (% Δ Int.)	Acet. (% Δ Int.)	Methy. (% Δ Int.)
3071 m ^a^	3070 (sc ^b^)	3065 (sc)	3073 (na)	3071 (na)
3008 sh	3008 (sc ^b^)	3002 (sc)	3010 (nc)	3008 (nc)
2940 m	2940 (na ^c^)	2937 (na)	2939 (–36)	2940 (–307)
2890 sh	2883 (sc ^b^)	2886 (sc)	- ^f^ (100)	2890 (–298)
2845 m	2846 (nc ^d^)	2843 (nc)	2846 (sc)	2845 (nc)
1662 s	1656 ^e^ (86)	1650 ^e^ (100)	1674 ^e^ (53)	1662 (94)
1621 sh	- ^f^ (100)	- ^f^ (100)	1622 (61)	1621 (100)
1597 vs	1602 (67)	1605 ^e^ (73)	1600 ^d^ (59)	1597 (65)
1508 vw	1508	- ^f^	1508	1508
1453 m	1453 (35)	1453 (51)	1454 (49)	1453 (–111)
1430 w	1429	1435 ^e^	1426	1430
1392 sh	- ^f^	- ^f^	1391	1392
1363 sh	1360	1351 ^e^	1367	1363
1334 m	1334 (69)	1334 (57)	1335 (52)	1334 (50)
1298 sh	- ^f^	- ^f^	1300	1298
1272 m	1271 (79)	1273 (64)	1274 (58)	1272 (58)
1226 vw	1222	1224	1222	1226
1192 w	1193 (33)	1190 (50)	1195 (36)	1192 (38)
1136 m	1135 (88)	- ^f^ (92)	1129 ^d,e^ (58)	1136 (78)
1089 w	1079 ^d,e^	- ^f^	1089	1089
1033 w	1032 (35)	1032 (47)	1034 (54)	1033 (–84)
975 vw	- ^f^	- ^f^	972	975
928 vw	927	929	936 ^d,e^	928
895 vw	884 ^d,e^	895	907 ^d,e^	895
811 sh	- ^f^	811	- ^f^	811
787 w	781 ^e^ (46)	789 (34)	792 ^e^ (70)	787 (54)
731 w	731 (41)	731 (14)	730 (44)	731 (–67)
637 vw	638	641	641	637
588 vw	- ^f^	- ^f^	607 ^e^	588
557 vw	562 ^e^	561	548 ^e^	557
534 vw	535	- ^f^	531	534
491 vw	484 ^e^ vw	- ^f^	490	491
457 vw	458	465 ^e^	461	457
361 w	368 ^e^ (42)	370 ^e^ (51)	366 (60)	361 ^e^ (73)

^a^ Note: vs. is very strong; s is strong; m is medium; w is weak; vw is very weak; and sh is shoulder. Band intensities are relative to other peaks in spectrum. ^b^ sc = Small change in intensity. ^c^ na = Not applicable because this band is expected to be minimally impacted and was used in spectra normalization. ^d^ nc = No change in intensity. ^e^ Band shifted. ^f^ Not detected.
